# Chronic pain is associated with disability: results from a large, population-based survey in South Africa

**DOI:** 10.1097/PR9.0000000000001417

**Published:** 2026-03-03

**Authors:** Murray McDonald, Peter R. Kamerman, Romy Parker

**Affiliations:** a Pain Management Unit, Department of Anaesthesia and Perioperative Medicine, Faculty of Health Sciences, University of Cape Town, Cape Town, South Africa; b Brain Function Research Group, Department of Physiology, School of Biomedical Sciences, Faculty of Health Sciences, University of the Witwatersrand, Johannesburg, South Africa

**Keywords:** Chronic pain, High impact, Disability, South Africa

## Abstract

**Introduction:**

Chronic pain significantly contributes to global disability, but data from South Africa and other low- and middle-income countries are limited.

**Objectives:**

We assessed the association between chronic pain (pain/discomfort ≥3 months) and disability using data from a large, population-based household survey. As an exploratory analysis, we also assessed for differences between individuals with high-impact chronic pain (HICP) (chronic pain with significant disability) to those with low-impact chronic pain (chronic pain with no disabilities) in demographics and health status variables.

**Methods:**

This cross-sectional, descriptive, secondary analysis used data collected in the 2016 South African Demographic and Health Survey, including 10336 adults. Disability was assessed using the Washington Group on Disability items. Statistical analyses were conducted using R v4.2.0 with the *survey* package.

**Results:**

Chronic pain prevalence was 18.3% (95% confidence interval [CI]: 16.9–19.7), with 27.8% (95% CI: 25.2–30.5) of individuals with chronic pain experiencing some form of disability. Chronic pain was associated with difficulties in cognition (adjusted odds ratio = 1.74 [95% CI: 1.41–2.14]) and mobility (AOR = 2.07 [95% CI: 1.70–2.52]), but not self-care (AOR = 1.07 [95% CI: 0.71–1.62]). The HICP group represented 1.7% (95% CI: 1.4–2.0) of the population and 9.2% (95%: 7.7–10.7) of those with chronic pain. Compared to the low-impact chronic pain group, the HICP group tended to be older and were more likely to be female, to receive a government grant, and to perceive their health as poor.

**Conclusion:**

Weak and moderately strong positive associations were found between chronic pain and greater cognitive and mobility disability, respectively.

## Abstract 2

Supplemental Digital Content is Available in the Text.

We show that chronic pain is associated with greater cognitive and physical disability, but not self-care, in a large population-based survey in South Africa.

## 1. Introduction

Chronic pain negatively affects physical function and participation in daily activities,^7,53,54^ including self-care.^17,52^ It also has a complex bidirectional negative relationship with psychological function, contributing to mood disturbances, anxiety, and cognitive decline.^51,66^ However, most reports on the disabling effects of chronic pain have been conducted in high-income settings. The findings from such settings may not fully apply to low- and middle-income settings where differences in disease burden (eg, communicable vs noncommunicable diseases), genetics, demographics, social norms, and healthcare systems may influence the impact of chronic pain.^29,30^

In this study, we investigated the association between chronic pain and disability across three domains: physical activity (impairment of walking or stair climbing), self-care (limited ability to wash and dress), and cognitive function (impaired ability to concentrate or remember) using data from a large, population-based survey in South Africa, an upper middle-income country that carries a significant burden of communicable (eg, HIV and tuberculosis infection) and noncommunicable disease (eg, diabetes mellitus and cardiovascular disease), has significant wealth and living standard disparities, including access to healthcare,^1,12,43^ but has a prevalence of chronic pain similar to that reported elsewhere.^31^

As an exploratory secondary aim, we investigated the concept of high-impact chronic pain (HICP), which refers to chronic pain that severely limits function, compared to low-impact chronic pain (LICP), which has less or no impact on function.^52,63,65^ Our aim was to determine whether individuals with HICP differed from those with LICP in demographics and health status variables.

## 2. Methods

### 2.1. Ethical approval

The study was approved by the South African Medical Research Council Ethics Committee (EC008-2/2015). Permission to use the data for a study titled, “Impact of pain in South Africa” was obtained from the Demographic and Health Surveys Program (https://dhsprogram.com).

### 2.2. Sampling

A total of 10336 adults aged 15 years or older were interviewed between April and November 2016 as part of the most recent South African Demographic and Health Survey funded by the South African government, the Global Fund to Fight AIDS, Tuberculosis and Malaria (Global Fund), the European Union, the United Nations Children's Fund, the United Nations Population Fund.^25^ This cross-sectional, descriptive national household survey aimed to provide estimates of demographic and health-related information for the country, achieving an 81% response rate for the adult health module (10336 out of 12717 eligible individuals). A brief outline of the sampling and interview processes is described below, with detailed descriptions in Supplement 1 (flowchart, http://links.lww.com/PR9/A386), Supplement 2 (sampling procedures, http://links.lww.com/PR9/A386), and Supplement 3 (fieldworker training procedures, http://links.lww.com/PR9/A386).

Eligible individuals were selected through a stratified 2-stage sampling procedure, detailed in Supplement 2 (http://links.lww.com/PR9/A386). The country was divided into primary sampling units (PSUs) using the Statistics South Africa Master Sample Frame based on the 2011 Census enumeration areas. In the first stage, 750 PSUs from 26 strata were selected with probability proportional to size. Within each PSU, a list of dwelling units was compiled to create the sampling frame for the second stage, which involved systematic selection of 20 dwelling units per PSU. In informal settlements, PSUs were segmented into groups of 20 dwelling units, with one segment randomly selected for sampling.

All households within selected dwelling units were eligible for interviews. In even-numbered dwelling units, all residents aged 15 years and older who were present the night before were eligible to complete the adult health module, excluding those unable to participate due to severe medical conditions or comprehension issues. The adult health module included key data for the analyses, such as demographic information, pain measures, disability assessments, and comorbidity evaluation. Modules were administered in-person (with no proxies) in one of South Africa's 11 official languages by 210 field workers organized into 30 teams. Each team consisted of a supervisor, three female interviewers, 1 male interviewer, 1 nurse, and 1 logistics officer/driver. All survey questions were rigorously forward and back translated and reviewed to ensure consensus before field deployment.

### 2.3. Outcome variables

Disability was assessed using questions sourced from the Washington Group on Disability Statistics developed under the United Nations Statistical Commission. These items are designed for large surveys and align with the International Classification of Functioning, Disability, and Health^25^ framework (https://www.washingtongroup-disability.com/question-sets/wg-short-set-on-functioning-wg-ss/). Six disability domains were assessed: problems with hearing, problems with seeing, problems with communicating, cognitive problems, problems with ambulation, and problems with self-care. We chose to use domains previously reported as being associated with pain, namely, cognitive problems,^48,52^ problems with ambulation,^17,53^ and problems with self-care.^17,52^ The questions used in the survey were worded as follows: (1) “do you have difficulty remembering or concentrating,” (2) “do you have difficulty walking a kilometre or climbing a flight of steps,” and (3) “do you have difficulty with self-care such as washing all over or dressing.” Each question was answered on a four-item Likert scale, ranging from no difficulty through to a complete loss of function. “Do not know” was a fifth option.

### 2.4. Variables of interest

Chronic pain was defined as having pain or discomfort either all the time, or on and off, for more than 3 months. High-impact chronic pain was defined as chronic pain (as defined above) accompanied by significant difficulty or complete inability in at least one of the following domains: remembering and/or concentrating, walking a kilometer and/or climbing stairs, washing and/or dressing. Low-impact chronic pain was defined as chronic pain (as defined) without any difficulty in any of the 3 domains. We extracted data on age, sex, literacy, government grant receipt (a needs-based monthly payment to qualifying South African residents),^25^ health insurance coverage, healthcare use, location and number of unique pain sites, self-perceived health status, and comorbid conditions (see below) from the dataset.

### 2.5. Covariates

Participant age (in years), sex (female or male), literacy (cannot read, partly able, able to read), government grant receipt (yes or no), residence location (urban or nonurban), and the presence or absence of other sources of disability were included as covariates in the statistical models (see *statistical analysis* for model details). Other sources of disability included: (1) having had a stroke; (2) having had a heart attack or angina/chest pains; (3) having chronic obstructive pulmonary disease; (4) having had, or currently having, cancer; and (5) having had tuberculosis (TB).

### 2.6. Statistical analyses

All statistical analyses were conducted using the R v4.2.0^59^ and the *survey* package,^40^ a package that factors in the complex sampling design and sampling weights in the calculation of summary and inferential statistics.

“Do not know” answers for the three disability questions were infrequent, accounting for less than 0.12% of answers. Instead of using listwise deletion of participant data when “Do not know” was provided as an answer, we took the conservative step of reclassifying all “Do not know” responses as having no disability.

For the primary question, we investigated the association between chronic pain and function in each of the three disability domains. Statistical models were generated for each disability domain using multiple ordinal regression with the Likert score as the dependent variable, chronic pain as the independent variable of interest, and age, sex, literacy, residence (urban vs non-urban), government grant receipt, and other potential sources of disability (TB, stroke, heart attack, chronic obstructive pulmonary disease, cancer) as covariates. All covariates were decided on a priori as factors that may affect the outcome and not following univariate screening of variables or using forward/backward selection on multivariable models. The 95% confidence intervals (CIs) of the odds ratios were used to interpret the direction, magnitude, and precision of the relationship between chronic pain and a disability.

To check for multicollinearity between model covariates, variance inflation factors were calculated for all models, and values of 5 or greater were taken to indicate collinearity. In the case of multiple ordinal regression models, the proportional odds assumption could not be directly tested for models constructed from a survey with a complex design. To overcome this problem, Likert scale cut-points were redefined into all possible binary combinations and binary logistic regression models, using a quasibinomial distribution family, were generated for each new binary cut-point. The estimates from each model, with their 95% CIs, were plotted and significant deviations in estimates were assessed by looking at the overlap between estimates and their CIs.

For the exploratory secondary question in those individuals with chronic pain, we compared people living with chronic pain and high disability (HICP; defined as the presence of chronic pain and a complete inability or a lot of difficulty in at least one of the domains of cognition, mobility, and self-care) to people living with chronic pain and no disability (LICP; defined as the presence of chronic pain without any difficulty in cognition, mobility, and self-care). We investigated whether age, sex, and number of unique pain sites (assessed using a “yes” or “no” question for a list of body regions: head/face, neck/shoulders, chest, abdomen, back, limbs, other) differed between the HICP and LICP groups. We also assessed whether having HICP (compared to LICP) was associated with worse health outcomes (self-perceived health status: poor vs average/good/excellent, receipt of government grants: “yes” or “no”, presence of healthcare seeking behaviour in the past 12 months: “yes” or “no”). As an exploratory analysis, we only performed univariate analyses. The relationship between HICP status and age and number of pain sites was assessed using linear regression, whereas the relationship between HICP status and binary variables was assessed by binary logistic regression models, using a quasibinomial distribution family. As an exploratory analysis, we do not report *P*-values, relying instead on interpretation of regression estimates (95% CIs) to assess the direction, magnitude, and precision of the relationships being assessed. No model checks were made or R^2^ values calculated for the linear regression models because data weighting and the use of stratification and clustered sampling can result in complex residual behaviours that cannot be interpreted using standard model check and fit metrics. All analysis scripts are available on Zenodo (DOI: 10.5281/zenodo.17182169), the data are available through the Demographic and Health Surveys Program (https://dhsprogram.com), and our reporting was aided by the STROBE guideline.^61^

## 3. Results

### 3.1. General description of the sample

Table 1 summarises the demographic and disease-related data for the entire cohort (N_Observed_ = 10336; pain prevalence = 18.3% [95% CI: 16.9–19.7]). In disability, about 7% of the cohort had difficulty concentrating, about 9% had difficulty walking or climbing stairs, and about 1.5% had difficulty caring for themselves. The average age was ∼39 years, and there were fewer males than females (∼40% male). Two-thirds of the cohort lived in urban areas, and literacy was poor (∼22% were unable or only partly able to read). Overall, ∼90% of all participants rated their health as average, good, or excellent. Consistent with these ratings, ∼88% had not sought healthcare in the past 12 months. Access to health insurance was low (16% had access). About 1% of the cohort had been diagnosed with cancer, having had a stroke, or having chronic obstructive pulmonary disease. Approximately 3% of the cohort had had a heart attack/angina, and ∼5% had being infected by TB.

**Table 1:** Demographic and disease characteristics for the full sample.

Characteristic	Full sample (N_Observed_ = 10336)
	Mean (95% CI)
Age (y)*	38.9 (38.3–39.6)

Characteristic	Full sample (N_Observed_ = 10336)
	Percent (95% CI)*
Had chronic pain	18.3 (16.9–19.7)
Had any difficulty remembering or concentrating†	7.1 (6.5–7.8)
Had any difficulty walking 1 km or climbing stairs†	8.8 (8.1–9.5)
Had any difficulty washing or dressing self†	1.4 (1.1–1.6)
Female‡	59.3 (58.1–60.4)
Lived in an urban area§	66.5 (64.3–68.7)
Literacy	
Cannot read	9.5 (8.6–10.4)
Partly able	13.1 (12.0–14.1)
Able to read	77.5 (75.9–79.0)
Perceived health status	
Poor	10.2 (9.4–11.0)
Average	30.8 (29.4–32.2)
Good	43.7 (42.2–45.2)
Excellent	15.3 (14.0–16.6)
Accessed healthcare in the past 12 mo	11.8 (10.8–12.7)
Had health insurance	16.0 (13.9–18.0)
Received government grant	20.1 (18.8–21.4)
Diagnosed with tuberculosis (TB)	5.5 (4.9–6.1)
Diagnosed with stroke	1.4 (1.1–1.7)
Diagnosed with heart disease	3.2 (2.7–3.6)
Diagnosed with cancer	1.1 (0.8–1.4)
Diagnosed with chronic obstructive pulmonary disease	1.5 (1.1–1.8)

* Age (y) percentiles: 0 (minimum) = 15, 25th = 24, 50th (median) = 35, 75th = 51, 100th (maximum) = 95.

† Any difficulty = complete inability, a lot of difficulty, or some difficulty with remembering or concentrating, walking a kilometer or climbing stairs, and/or washing or dressing themselves.

‡ Sex was recorded as a binary variable (male or female).

§ Original categories consisted of urban, rural, and traditional places of residence, but we combined the rural and traditional categories into a single “non-urban” category.

### 3.2. Primary outcomes

The results of the primary analyses comparing chronic pain and disability are presented in Table 2.

**Table 2:** Chronic pain associations with disability.

Characteristic	Chronic pain		Adjusted odds ratio (95% CI)*‡	McFadden Pseudo-R* (%)†
	No (N_Observed_ = 8389)	Yes (N_Observed_ = 1947)		
	Percent (95% CI)*			
Difficulty remembering or concentrating			**1.74** (**1.41–2.14**)	16.0
No difficulty	94.6 (94.0–95.2)	85.1 (83.1–87.2)		
Some difficulty	4.2 (3.7–4.8)	11.8 (10.0–13.5)		
A lot of difficulty	1.0 (0.8–1.3)	2.8 (2.1–3.5)		
Cannot at all	0.1 (0.1–0.2)	0.3 (0.1–0.5)		
Difficulty walking 1 km or climbing stairs			**2.07** (**1.7–2.52**)	24.0
No difficulty	93.7 (93.1–94.4)	80.0 (77.6–82.3)		
Some difficulty	4.3 (3.8–4.8)	13.2 (11.2–15.1)		
A lot of difficulty	1.7 (1.4–2.0)	6.2 (5.0–7.3)		
Cannot at all	0.3 (0.2–0.4)	0.8 (0.4–1.1)		
Difficulty washing or dressing self			**1.07** (**0.71–1.62**)	21.3
No difficulty	98.9 (98.7–99.2)	97.4 (96.7–98.1)		
Some difficulty	0.7 (0.5–0.9)	1.7 (1.1–2.2)		
A lot of difficulty	0.3 (0.1–0.4)	0.5 (0.2–0.9)		
Cannot at all	0.1 (0.1–0.2)	0.4 (0.1–0.6)		

* Reference level: no chronic pain.

† Percentage of model deviance explained compared to the null model.

‡ Adjusted for age; sex; literacy; government grant receipt; urban/nonurban residence; diagnosed with chronic obstructive pulmonary disease; diagnosed as having had heart disease (heart attack and/or angina pectoris); diagnosed with having, or having had, cancer; diagnosed with having had a stroke; diagnosed with having had tuberculosis.

### 3.3. Model checks

All models had variance inflation factors less than 5 (ie, no indication of multicollinearity between variables in the models). Visual checks of the proportional odds assumption of ordinal regression models indicated no serious deviations from the assumption (Supplement 4, http://links.lww.com/PR9/A386).

### 3.4. Cognition

Chronic pain was associated with higher odds of having a cognitive disability compared to those without chronic pain (adjusted odds ratio = 1.74 [95% CI: 1.41–2.14]). However, with the likelihood of residual confounding and inherent measurement error, we interpreted this effect as being weak. Most participants, regardless of chronic pain status, reporting “no difficulty remembering or concentrating,” with few participants in either group reporting “a lot of difficulty” or “could not remember or concentrate at all.”

### 3.5. Mobility

The findings for the ability to walk or climb stairs were similar to those for cognitive function, such that chronic pain was associated with greater disability (adjusted odds ratio = 2.07 [95% CI: 1.70–2.52]); the relationship was moderately strong. Again, the vast majority, regardless of pain presence, reported “no difficulty walking or climbing stairs.”

### 3.6. Self-care

Almost all participants (∼98%), regardless of chronic pain status, reporting no difficulty with self-care. Consistent with these data, the point estimate indicated a very weak, positive association (adjusted odds ratio = 1.07), but the 95% CI for the adjusted odds ratio was ambiguous, ranging from 0.71 to 1.62, and based on the likelihood of residual confounding and inherent measurement error, we interpreted this effect as being very unlikely to be positive.

### 3.7. Secondary outcomes

The results of the secondary analyses, which assessed differences between the HICP group and the low-impact chronic pain group (chronic pain with no disabilities) in demographics and health status, are shown in Table 3.

**Table 3:** Exploratory analysis of variables associated with having high-impact chronic pain.

Variable	LICP* (N_Observed_ = 1350)	HICP† (N_Observed_ = 234)	Estimate (95% CI)‡
	Mean (95% CI)		Beta coefficient
Age (y)	41.7 (40.2–43.1)§	63.8 (61.3–66.3)‖	22.2 (19.3–25.0)
No. of unique pain sites	1.3 (1.2–1.3)	1.6 (1.5–1.8)	0.4 (0.2–0.5)

Variable	LICP* (N_Observed_ = 1350)	HICP† (N_Observed_ = 234)	Estimate (95% CI)‡
	Percent (95% CI)		Odds ratio
Female	61.0 (56.8–65.0)	77.1 (71.3–82.8)	2.16 (1.49–3.13)
Received a government grant	19.9 (17.1–22.9)	70.6 (63.2–78.0)	9.62 (6.49–14.25)
Accessed healthcare in the past 12 mo	16.1 (13.5–18.7)	21.7 (16.0–27.4)	1.44 (0.98–2.13)
Perceived health status was poor	17.1 (14.5–19.7)	54.0 (46.2–61.7)	5.69 (3.93–8.23)

* Low-impact chronic pain (LICP) = chronic pain without any difficulty remembering and/or concentrating, walking a kilometre and/or climbing stairs, and/or washing or dressing themselves.

† High-impact chronic pain (HICP) = chronic pain with complete inability or a lot of difficulty with remembering and/or concentrating, walking a kilometer and/or climbing stairs, and/or washing or dressing themselves.

‡ Reference level: LICP.

§ LICP age (y) percentiles: 0 (minimum) = 15, 25th = 27, 50th (median) = 39, 75th = 56, 100th (maximum) = 95.

‖ HICP age (y) percentiles: 0 (minimum) = 20, 25th = 54, 50th (median) = 66, 75th = 76, 100th (maximum) = 95.

Those with HICP represented 1.7% (95% CI: 1.4–2.0) of the total cohort and 9.2% (95%: 7.7–10.7) of those with chronic pain. Compared to individuals with LICP, those with HICP were more likely to be female (HICP ∼75% female; LICP ∼60% female). Those with HICP were also older by about 20 years on average (large effect size—beta coefficient—and narrow CI), and about half rated their health as being poor (∼3 fold higher than the LICP group; the effect size—odds ratio—was large, and although the CI was wide, the lower limit was still consistent with a large effect size). Most (70%) of the HICP group received some form of government grant (almost 4-fold higher than the LICP group; the effect size—odds ratio—was very large, but precision of the estimate was low). On average, people with HICP had greater number of unique pain sites than people in the LICP group, but the magnitude of the effect size—beta coefficient—was marginal. Moreover, despite substantially more people in the HICP group rating their health as being poor compared to people in the LICP group, healthcare seeking behaviour was about the same in the 2 groups (small effect size—odds ratio—with ambiguous CI).

## 4. Discussion

### 4.1. Chronic pain and disability in South Africa

Using data from a large, population-based survey in South Africa, we investigated the association between chronic pain and disability across three domains: physical activity, cognitive function, and self-care. Our secondary aim was to determine whether individuals with HICP differed from those with LICP in selected demographic and health status variables. For our primary objective, we found weak and moderately strong positive associations between chronic pain and greater cognitive and mobility disability domains, respectively, but not with self-care. For our exploratory secondary objectives, we found that compared to the LICP group, the HICP group were more likely to be older, female, rate their health as “poor,” and receive a government grant, yet they were no more likely to use healthcare.

Previous South African studies have shown that chronic pain impairs function and limits activity in people attending primary care,^53^ HIV clinics,^20^ palliative care clinics,^9,16^ and in people suffering from conditions such as inflammatory arthritis.^5^ The data from this study show that this relationship is present at a population level for disability in cognition and mobility. However, caution should be taken when interpreting the cognition and mobility results. First, for both these domains, very few people, irrespective of whether they had chronic pain, had severe disability. That is, when disability was present, it tended to be mild to moderate in severity. Second, the relationship between chronic pain and disability may be bi-directional, as disabling conditions can also lead to chronic pain, so cause and effect cannot be confirmed through these associations.^11,19,51^

Cognitive function encompasses several domains, including executive function, language processing, motor skills, attention, and memory.^32^ Previous studies have associated cognitive impairment with chronic pain,^32,48^ suggesting it may be a risk factor for cognitive decline with age.^66^ Chronic pain may impact cognitive function through mechanisms such as competing resources, neuroplasticity, and/or dysregulated neurochemistry.^48^ In this study, cognitive impairment was assessed only in attention and memory, which does not provide a comprehensive measure of cognition. In addition, sleep and depression, which are notable mediating or moderating factors in this relationship,^34^ were unavailable in this dataset.

The association between chronic pain and reduced physical function is well established in international populations^7,54^ and has been observed in local primary care, rural, and urban populations.^15,26,53^ In this study, physical function was measured by assessing walking and stair-climbing abilities. Although these activities may reflect the higher occurrence of limb and back pain in our sample, walking and stair-climbing might not necessarily be as affected by pain in other areas e.g. neck pain. A more comprehensive measure of physical function may have yielded a higher level of dysfunction, and thus affected the association with chronic pain. In addition, this relationship may be moderated by factors such as physical fitness, depression, anxiety, and analgesic use.^6,37–39^

Previous studies have identified self-care difficulties as common consequences of chronic pain.^17,52^ A U.S. study found self-care limitations only in those with HICP, who had nearly three times the self-care disability rate as our sample (∼3.7% vs ∼1.4%).^52^ Similarly, a Spanish study found chronic pain affected washing/dressing least among 13 activities.^17^ The lack of association between chronic pain and self-care limitations in South Africa may be due to underreporting or a true lack of effect. Underreporting could stem from perceived stigma associated with being sick and potential causes of that illness (eg, HIV infection). Alternatively, a true lack of association might be due to the low prevalence of self-care limitations, the perceived importance or prioritisation of other activities, or having sufficient help with self-care tasks from family or carer support.^24,28,41,45,55,56,65^

South Africans with chronic pain were more likely to seek healthcare, particularly from public facilities, compared to those without chronic pain. Painful conditions are among the most common reasons for seeking medical care both globally^22^ and in South Africa.^33^ If chronic pain is driving increased demand for healthcare services, it could substantially burden the public healthcare system. This may have significant economic consequences in an increasingly ageing population,^14,36^ necessitating health system strengthening to meet and manage the demand.^8,57^

### 4.2. High-impact chronic pain in South Africa

We defined HICP as chronic pain with significant cognitive, mobility or self-care difficulties, and found that ∼1.7% of South Africans experience HICP. Other countries have also attempted to define HICP,^3,17,62^ but a consensus definition remains elusive,^18^ making comparisons challenging due to different methodologies. For example, in the United States, HICP prevalence was estimated at 8% using a definition of pain on most days for the last 6 months with significant limitations in life or work activities.^13^ In Saudi Arabia, the prevalence was 4% using a definition of chronic pain affecting activities of daily living for 3 months or more, with pain severity of at least 7 on a 0 to 10 scale. In Spain, a prevalence of 2.4% was found using a definition of chronic pain on at least 4 to 5 days per week for 3 months and cluster analysis of 13 impairments, limitations, and restrictions.^17^

Due to survey limitations, we were unable to analyse the relationship between HICP and life role restrictions such as employment, interpersonal relationships, or community engagement. However, compared to people with chronic pain but without disability (LICP), our HICP group shows a strong association with poor self-perceived health, and a strong association with receiving financial support from the government via a social grant. Therefore, we consider our definition and analysis a valid representation of HICP.^18^

South Africans with HICP are more likely to be older and female than those with chronic pain who are not disabled. The association between age and HICP aligns with studies from the United States,^52^ Saudia Arabia,^3^ and Spain,^17^ possibly due to the relationship between age and disability.^58,64^ However, although the relationship between female sex and HICP was found in Saudi Arabia,^3^ it was not observed in the United States,^52^ or Spain.^17^ This discrepancy could be due to methodological differences or societal and gender role factors in disability.^10,46,47^

Interestingly, we found no meaningful association between number of unique pain sites and HICP, aligning with some studies^17^ but in disagreement with others.^17,63^ This discrepancy may be due to methodological differences or indicate that a single pain location can cause significant disability.

In our sample, those with HICP were more likely to rate their health as “poor” compared to others with chronic pain but were no more likely to seek healthcare in the previous 12 months, which contrasts with international data.^3,17,52^ Our study and others suggest that South Africans with chronic pain show higher levels of care seeking.^33,44^ However, South Africans with disability (especially in cognition and mobility domains) exhibit higher levels of depression and barriers to healthcare,^23,50^ which might explain the lower than expected healthcare usage in our HICP group. This could also be due to a lack of access to care, availability of care, and/or transport^2,21,60^ especially considering that they were older and more than 50% were from nonurban areas.^60^ Further investigation into the care needs and barriers of this group should be a research priority.

## 5. Limitations

We used a metric for disability and HICP that was relatively narrow in domain and cut-offs that were relatively strict to represent the people living with disabilities. Using this restricted definition may underrepresent the extent of disability/HICP in this population,^23,27,35,42^ which would affect the generalisability of our findings to other populations.

These data were collected in 2016 and published in 2019 thus may not reflect the current state of pain-related disability. However, the burden of noncommunicable disease^1^ and socioeconomic circumstances are unlikely to have changed remarkably^4,49^ and may reasonably reflect the current state of pain-related disability.

## 6. Recommendations

This research shows that chronic pain is significantly associated with disability in a large, nationally representative population of an upper middle-income country. We recommend that chronic pain should be increasingly included in the national healthcare agenda and encourage further research into the relationship between chronic pain and disability, particularly in LMIC. The concept of HICP appears to be relevant to the South African context and warrants further conceptual refinement and investigation into its utility. We also recommend that any future SADHS or similar survey monitor pain treatments and barriers to healthcare to better understand how chronic pain is managed in South Africa.

## Disclosures

The authors have no conflict of interest to declare.

## Supplemental digital content

Supplemental digital content associated with this article can be found online at http://links.lww.com/PR9/A386.
